# Foliage Intensity is an Important Cue of Habitat Location for *Empoasca onukii*

**DOI:** 10.3390/insects11070426

**Published:** 2020-07-09

**Authors:** Lei Bian, Xiao Ming Cai, Zong Xiu Luo, Zhao Qun Li, Zong Mao Chen

**Affiliations:** 1 Tea Research Institute, Chinese Academy of Agricultural Science, 9 Meiling South Road, Xihu District, Hangzhou 310008, China; bianlei@tricaas.com (L.B.); cxm_d@tricaas.com (X.M.C.); luozongxiu@tricaas.com (Z.X.L.); zqli@tricaas.com (Z.Q.L.); 2 Key Laboratory of Tea Biology and Resource Utilization, Ministry of Agriculture, 9 Meiling South Road, Xihu District, Hangzhou 310008, China

**Keywords:** compound eye, *Empoasca onukii*, foliage color, habitat location, intensity, vision

## Abstract

For many herbivorous insects, vision is more important than olfaction in the prealighting stage of host habitat location. Tea leafhoppers, *Empoasca onukii* (Hemiptera, Cicadellidae), are serious pests that preferentially inhabit the tender leaves of tea plants across China. Here, we investigated whether tea leafhoppers could distinguish foliage colors associated with different leaf ages and use this visual cue to guide suitable habitat location from short distances. Similar to honeybees, the adult *E. onukii* has an apposition type of compound eye, and each ommatidium has eight retinular cells, in which three spectral types of photoreceptors are distributed, with peak sensitivities at 356 nm (ultraviolet), 435 nm (blue), and 542 nm (green). Both changes in spectral intensity and hue of reflectance light of the host foliage were correlated with varying leaf age, and the intensity linearly decreased with increasing leaf age. Behavioral responses also showed that adult *E. onukii* could discriminate between the simulated colors of host foliage at different leaf ages without olfactory stimuli and selected the bright colors that strongly corresponded to those of tender leaves. The results suggest that, compared with the spectral composition (hue), the intensity of light reflectance from leaves at different ages is more important for adult leafhoppers when discriminating host foliage and could guide them to tender leaves at the top of tea shoots.

## 1. Introduction

*Empoasca onukii*, a small hemipteran insect, is a serious pest of tea plants. The distribution of *E. onukii* in tea shoots is very regular, with active adults inhabiting the first to fourth leaf below the bud in growing tea shoots [1] and with most female adults preferring the second leaf to complete oviposition [2,3]. Host habitat location by most herbivorous insects is mediated by the interplay between chemical and visual cues [4,5,6]. However, for members of the genus of *Empoasca*, most species detect suitable habitats based mainly on visual cues. For example, chemical stimuli from the foliage of cotton could only be detected by *Empoasca devastans* from 1 cm, but visual stimuli were effective at up to 3.6 m (i.e., vision is the only option for *E. devastans* from a range from 1 cm to 3.6 m) [7]. Odors from the host were unable to attract *E. vitis* over long distances (>20 cm), across which visual cues (color) are the dominant information for *E. vitis* in selecting habitat [8]. Visual cues may play an important role in habitant location at a distance for *E. vitis* adults.

The visual cues could include color parameters such as hue (H, dominant wavelength of reflected light), saturation (S, spectral purity of reflected light), and brightness (B, intensity of perceived reflected light) and some spatial information on shape, size, distance, and motion [6]. Numerous action spectra studies have shown that Cicadellidae insects have positive responses to different colors [9,10,11], which implies that these leafhoppers have well-developed vision and a high sensitivity to color. *Limotettix vaccinii* adults prefer green, and *Scaphytopius magdalensis* adults exhibit a preference for yellow [10]. *E. onukii* adults can also be attracted to a series of colors near yellow or green hues [9]. Insect attraction to different colors has been extensively studied in hemipteran insects, but studies in sensory physiology of hemipteran eyes have remained sparse, possibly because of their small size [6,12]. True color vision requires a comparison of signals from at least two types of photoreceptors with different spectral sensitivities [13,14]. The photoreceptors in the compound eyes of winged insects (Pterygota) typically have three basic spectral sensitivity ranges, i.e., the ultraviolet (UV, ~350 nm), blue (~440 nm), and green (~530 nm) parts of the spectrum [13,15].

The visual physiological basis of color discrimination has been extensively studied in pollinating insects [14,16,17] and in insects harmful to fruit crops [18], and the target objects often appear to strongly contrast with the green background. For example, the flowers and cones of Norway spruce (*Picea abies*) provide detectable achromatic contrasts with the background of needles for the spruce seed moth (*Cydia strobilella*) [15]. However, the spectral compositions of foliage under diffuse lighting conditions are generally consistent over a wide range of species [6,19]. The fresh tea leaves below the bud also appear to have green hues that are similar to the rest of the leaves in the same branch. It was reported that, in some aphids, discrimination between portions of plants in particular physiological stages may be at least partly accomplished based on differences in intensity of reflected light [6]. If *E. onukii* adults are to accurately find a suitable habitat leaf in a tea shoot by using color cues, a strong ability to detect foliage spectral contrasts is necessary.

In this study, the ability of *E. onukii* adults to discriminate host foliage colors was discussed. Because of the lack of visual physiological information on *E. onukii*, we first identified mRNAs encoding visual opsins and the basic internal structure of the ommatidia. In addition, the spectral sensitivity of the compound eye was measured by recording electroretinograms (ERGs). Then, the reflectance spectral curves and color cues of foliage in growing tea shoots of different ages were measured. Finally, the ability of *E. onukii* adults to respond to different simulated colors was determined.

## 2. Materials and Methods

### 2.1. Insects and Plants

*Empoasca onukii* adult leafhoppers and host samples of the “Longjing 43” tea cultivar were obtained in May and June from the Tea Research Institute at the Chinese Academy of Agricultural Sciences, Hangzhou, China.

The insects were reared on “Longjing 43” cultivar tea plants in the laboratory at 25 ± 2 °C with 70 ± 3% relative humidity, under a 14-h light/10-h dark photoperiod. All female and male adults used in the experiments were 2–4 days old. The tea samples of the “Longjing 43” cultivar were actively growing, without pests or disease affecting them. Before the experiments, all tea samples were reared in water at 25 ± 2 °C with 70 ± 3% relative humidity, under a 14-h light/10-h dark photoperiod.

### 2.2. Datamining, Gene Cloning, and Phylogenetic Analysis

Based on the prior transcriptome analyses on the *E. onukii* head (Sequence Read Archive experiment accession number: SRP091412; BioProject accession number: PRJNA347531) [20], opsin proteins were screened from the databases along with their functional annotations. The de novo transcriptome was assembled using the short-read assembly program Trinity v.2.1.1. Unigenes larger than 200 bp were used as queries to search the following protein databases with the BLASTX online tool: Nr (e-value < 10^−5^), Nt (e-value < 10^−5^), Pfam (e-value < 0.01), KOG/COG (e-value < 10^−3^), Swiss-Prot (e-value < 10^−5^), KEGG (e-value < 10^−10^), and GO (e-value < 10^−6^). Proteins with the highest sequence similarity to the proteins encoded by the unigenes were retrieved from the databases along with their functional annotations. The nucleotide sequences of the opsin genes were then confirmed by gene cloning and sequencing. Total RNA was first extracted from *E. onukii* heads and reverse transcribed to generate cDNA, which was used as the PCR template for confirming the opsin gene sequences. The full-length target genes were amplified by PCR using ExTaq DNA polymerase (TaKaRa, Dalian, China) and gene-specific primers (Table S1). The PCR program was as follows: 94 °C for 4 min; 35 cycles of 94 °C for 30 s, 55 °C for 30 s, and 72 °C for 40 s; and 72 °C for 10 min. Amplicons were analyzed by 1.5% agarose gel electrophoresis and then subcloned into the pGEM-T Easy vector for sequencing. The amino acid sequences of the opsin proteins were used along with proteins from 10 other insect species to construct the phylogenetic tree. The amino acid sequences of all opsin proteins were aligned using Mafft (version 7.311), and the protein weight matrix used Blosum62 and then were analyzed by the maximum likelihood method using the Poisson correction distance, nearest-neighbor interchange method, very strong branch swap filter, and 1000 bootstrap replicates with MEGA 6.0 for the phylogenetic tree construction [21]. The transcript abundances of the target opsin genes were calculated based on the reads per kilobase per million mapped reads (RPKM) method [22]. The RPKM values were calculated using the following equation:

(1) $$\mathrm{RPKM}(A)=\frac{C\times 10^{6}}{\frac{N\times L}{10^{3}}}$$

where RPKM (A) refers to transcript A, *C* is the number of reads uniquely aligned to transcript A, *N* is the total number of fragments uniquely aligned to all transcripts, and *L* is the number of bases in transcript A.

### 2.3. Histology

Internal fine structure of the ommatidia was observed with light and electron microscopy. After 4 h of light adaptation, the tea leafhoppers were decapitated and the heads was then immediately fixed in 4% paraformaldehyde in 0.1 M phosphate-buffered saline (PBS; pH 7.2–7.4). For light microscopy, the samples were then dehydrated in gradients of 50–100% ethanol and immersed in wax for 4 h. The paraffin sections (4 μm) were cut on a microtome (Slee Cut-4062, Slee Medical GmbH, Mainz, Germany) and then placed on glass slides. After hematoxylin-eosin staining and mounting, the samples were observed with a regular optical microscope (Olympus BX41, Tokyo, Japan) with a ×50 lens and a digital camera (Canon, Tokyo, Japan). For electron microscopy, the samples were post-fixed in 1% OsO4, buffered in 0.1 M PBS for 2 h at room temperature, and then rinsed three times and dehydrated in gradients of 50–95% ethanol and 100% acetone. After being pretreated three times in pure propylene oxide, the samples were passed through a series of propylenoxid/Epon mixtures (3:1, 2:1, 1:1, 1:2, 1:3, and pure Epon), embedded in pure Epon (Serva, Heidelberg, Germany), and hardened at a temperature of 70 °C for 24 h. The ultrathin sections were stained with 2% aqueous uranyl acetate for 15 min and with Reynolds’ lead citrate for 5 min and then observed with a transmission electron microscope (Hitachi H-7650, Tokyo, Japan).

### 2.4. Electroretinography Recordings

The spectral sensitivity of adult tea leafhoppers was determined by recording ERGs. A series of monochromatic stimuli were provided by a 150 W xenon arc lamp through diverse narrow-band interference filters (300–700 nm, bandwidth 10 nm, Andover Corp., Salem, NH, USA). The monochromatic light, which illuminated the entire body of the leafhopper, was directed via an optical fiber when a shutter was opened.

Based on the method of Wakakuwa et al. [12], the leafhopper, with notum upwards, was fixed with glue onto a black plastic board. In a dark Faraday cage, an electrolytically sharpened tungsten electrode inserted into the abdomen served as the reference electrode. The tip of a capillary glass microelectrode filled with tap water touched a small amount of conductive paste at the dorsal surface of the compound eye. Prior to recording ERGs, the leafhopper was dark-adapted for 30 min and then stimulated with flashes of 100-ms duration, presented 5 s apart. The maximum quantum flux of each tested wavelength was adjusted to approximately 10^11^·photons·cm^−2^·s^−1^ by the light source regulator and neutral-density filters (Andover Corp., Salem, NH, USA). The spectral sensitivity was measured five times for each sex, from 17:00 to 20:00 during a day, to avoid the effect of circadian rhythms [23], with wavelengths alternating from 300 to 700 nm; then, the procedure was repeated in the reverse order, yielding ten spectral scans. The intensity output of the monochromatic light at each wavelength was calibrated by series of neutral-density filters. Over a 4-log unit intensity range at each wavelength, the response amplitude was transformed to equivalent intensities (log *I*) through the Naka–Rushton function, *V*/*V_max_* = *I^n^*/(*I^n^* + *K^n^*), where *V* represents the response amplitude, *V_max_* represents the maximum response amplitude, *I* is the stimulus intensity, *K* represents the stimulus intensity eliciting 50% of *V_max_*, and *n* is the exponential slope. Finally, the spectral sensitivity was converted by taking the reciprocal of all calculated stimulus intensities [24].

### 2.5. Reflectance Spectra of Host Foliage

To figure out the variation in spectral composition and intensity of tea foliage of different ages, the method of Johnsen was used as a reference [25]. The diffuse reflectance spectra from the obverse surface of single tea leaves were measured in a darkened room with a fiber optic spectrometer system constructed from a spectrometer (PG2000 pro, Ideaoptics, Shanghai, China), a light source (300–1100 nm, Ideaoptics), a Y-type coupling optic fiber (FIB-Y-600-TA-DUV, Ideaoptics), and a holder that fixed the distance and angle between the fiber and foliage. The distance from the leaf surface to the fiber was kept at 5 mm. The diffuse light from the blade surface, which was vertically irradiated by light transmitted through six peripheral fiber cores (diameter 600 μm), was collected by the center fiber core (diameter 600 μm) and then quantified in the spectrometer.

**Figure 1:**
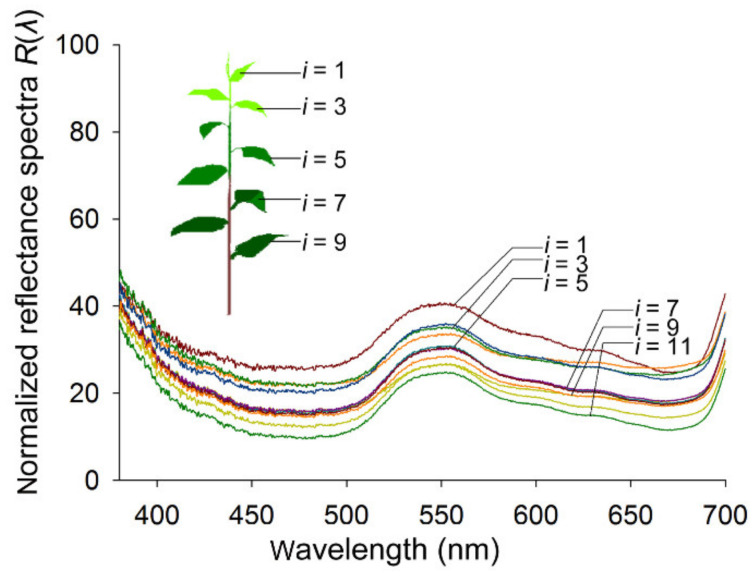
The normalized reflectance spectra *R*(λ)*_i_* of tea leaves: The inset diagram is of a tea shoot showing the tea foliage of different leaf ages, numbered with *i* (*i* = 1–11) from the top to bottom. On the same side of the leaf, four sampling points were selected in each leaf. Obverse sides of the leaves from a total of six repeated shoots were measured. Therefore, each *R*(λ)*_i_* was the average of 24 measured spectra from each type (*i*) of foliage.

From the top to the bottom of a tea shoot, differently aged leaves below the bud were numbered (*i* = 1, 2…11, Figure 1) [1], and their reflectance spectra were measured. On the same side of the leaf, four sampling points were selected. The obverse sides of the leaves from a total of six different shoots collected from different tea trees were measured. The spectral data were recorded with a minimum integration time of 50 μs and an average of three measurements for each spectrum, which was analyzed online in Morpho 3.2 software (Ideaoptics) and then converted into normalized reflectance spectra (*R_i_*(*λ*)) [25,26]:

*R_i_*(*λ*) = *S*(*λ*)*L*(*λ*)^−1^100% (2)

where *i* is the number of leaves of different ages, *λ* is the wavelength (from 300 to 700 nm), *S*(*λ*) is the reflectance spectra from the sample, and the *L*(*λ*) is the reflectance spectra from a white standard (STD-WS, Ideaoptics). Then, the chromatic contrast and achromatic contrast (intensity) of these leaves were analyzed.

It is known that proportional spectral changes across the entire spectrum will not change the hue of a color. For different color hues, particular parts of the spectrum would have to change their contribution. Therefore, to determine the variation in spectral composition of tea foliage (*i* = 1, 2…11), the Pearson correlation coefficients for all *R_i_*(*λ*)s were first calculated using SPSS 14.0 (SPSS, Inc., Chicago, IL, USA). High coefficients (0.8–1) indicated that there were high correlations among variables. Then, linear regressions among *R_i_*(*λ*)s were conducted to estimate how the hue of leaf changed.

To analyze achromatic contrast (intensity), the normalized reflectance intensity (*I_i_*) of each leaf was as follows:

(3) $$I_{i}=\int_{300}^{700}R_{i}(\lambda )d\lambda$$

where *i* is the number of leaves of different ages, *λ* is the wavelength (from 300 to 700 nm), and *R*(*λ*) is the normalized reflectance spectra from the sample. Then, the linear regressions between *I_i_* and *i* were analyzed to determine the variation in intensity of these foliage.

### 2.6. Behavioral Experiments

The colors of tea leaves (*i* = 1, 2…11) below the bud were measured with a color variation meter (Konica Minolta CM-700d, Tokyo, Japan) to obtain the color parameters (HSB color mode) from a white standard. On the obverse side of each leaf, on a total of six different branches, four sampling points were selected and measured. The means of these color parameters (*H*, *S*, and *B*) were then used for the simulated colors of tea foliage in behavioral experiments. A behavioral observation box was used to test the “first choice” of different simulated colors by *E. onukii* adults [9]. The box, constructed of plexiglass, was divided by four opaque internal panes into four vertical trapezoidal columns of equal volume. The top and bottom square panes of the box were opaque black, but the four sides were transparent. Through the four transparent sides, the leafhoppers released at the center of the box could initially see four liquid crystal displays (LCDs; Acer V193W, Nanjing, China), which were controlled by computers and provided simulated colors. The display brightness of all LCDs was set to 50%. Leafhoppers that were attracted to a particular simulated color would fly toward the corresponding LCD and be trapped on the inner side in glue.

Behavioral experiments were conducted from 17:00 to 20:00, which is the flight activity peak of *E. onuki*. The white color (control) and all 11 simulated colors (*i* from 1 to 11) were presented in a randomized order on the four external LCDs, i.e., each LCD contained three equal-area rectangles of different colors side-by-side. The male and female adult leafhoppers were determined separately. Each experiment for males or females lasted 20 min, after 10 min of dark preprocessing, and was repeated five times. The total number of tea leafhoppers was 60 in each replication. When the test ended, the leafhoppers trapped by each color were counted (*N_i_*). Significant differences in the numbers of trapped male and female leafhoppers for the same color were calculated using independent-samples *t*-tests. Significant differences among the numbers of trapped leafhoppers of the same sex for different colors were calculated by one-way analysis of variance followed by Fisher’s LSD multiple comparison test using SPSS 14.0. In addition, Pearson correlation coefficients and the linear regressions between *N_i_* and the normalized reflectance intensity (*I_i_*), and the two-color parameters (*S_i_* and *B_i_*) were included to analyze their relationships.

### 2.7. Discrimination of Host Foliage Color

Both chromatic and achromatic contrast existed among host foliage, but their respective weight in the discrimination of foliage colors by *E. onukii* needs further analysis. Based on the physiological results, whether the foliage colors (chromatic contrast) could be discriminated by the compound eyes of *E. onukii* adults is estimated with the “receptor-noise-limited color opponent model” [15,27,28].

First, the color stimulus is defined by photoreceptor quantum catches:

(4) $$Q_{j}=\int_{300}^{700}q_{j}(\lambda )R_{j}(\lambda )I_{j}(\lambda )d\lambda$$

where *j* is the type of photoreceptor (*j* = UV (S), blue (M), or green (L)), *λ* is the wavelength (from 300 to 700 nm), *q_j_* is the sensitivity of photoreceptor type *j*, *R_j_*(*λ*) is the reflectance spectra of the foliage, and *I_j_*(*λ*) is the illumination spectra. Then, the contrast for each photoreceptor channel is as follows:

(5) $$\Delta f_{j}\;=\;\ln\Delta Q_{j}$$

The chromatic contrast of two different colors is as follows:

(6) $$\Delta S\;=\;\sqrt{\frac{\omega_{S}^{2}{\left(\Delta f_{L}-\Delta f_{M}\right)}^{2}+\omega_{M}^{2}{\left(\Delta f_{L}-\Delta f_{S}\right)}^{2}+\omega_{L}^{2}{\left(\Delta f_{M}-\Delta f_{S}\right)}^{2}}{{\left(\omega_{S}\omega_{M}\right)}^{2}+{\left(\omega_{S}\omega_{L}\right)}^{2}+{\left(\omega_{M}\omega_{L}\right)}^{2}}}$$

where ω is the Weber fraction determined by noise in each photoreceptor channel. The value of ω is set in term of honeybees [28], which have a certain similarity with the ommatidium structure of leafhoppers [12]. When ΔS is <2.3, color discrimination is considered impossible for honeybees [29].

It is worth mentioning that this model just predicts chromatic contrast but ignores intensity [15], which is also a principal stimulus eliciting alightment on living plants and is a more variable foliage parameter than is spectral composition [6]. Therefore, the Michelson contrast of foliage was calculated as follows [15,30]:

(7) $$C=\frac{Q_{\max}-Q_{\min}}{Q_{\max}+Q_{\min}}$$

where *Q*_max_ and *Q*_min_ are the stimulus surfaces of tea foliage yielding the highest and lowest quantum catches in the wavelength range from 300 to 700 nm, respectively. Again referring to honeybees, when *C* is <0.08, intensity discrimination is considered impossible [15].

## 3. Results

### 3.1. Opsins and Compound Eye Structure

**Figure 2:**
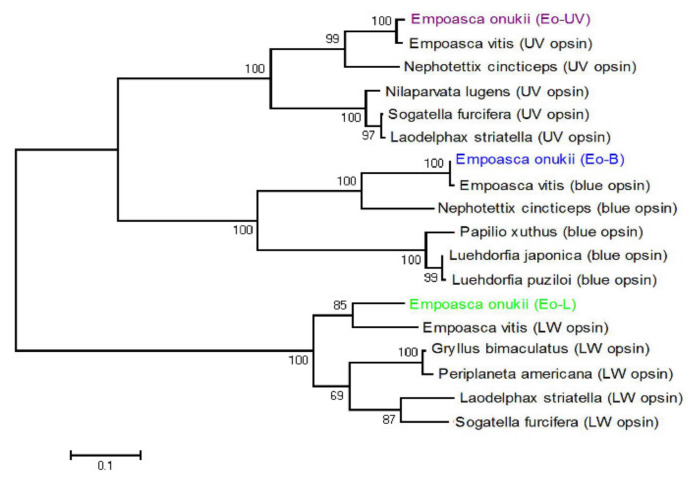
Phylogenetic relationships among three types of opsin from 10 species: *Empoasca onukii* express three types of opsin, ultraviolet (UV), blue (B), and long wavelength (LW) absorbing opsins, colored in violet (*Eo*-UV, short wavelength clade), blue (*Eo*-B, middle-wavelength clade), and green (*Eo*-L, long-wavelength clade), respectively.

Phylogenetic analysis showed that there are three types of opsin amino acid sequences clustered in the UV, blue, and long wavelength absorbing opsin clades of several insects (Figure 2). Therefore, the three visual opsins were termed *E. onukii* UV, B, and LW (GenBank accession numbers MH844497–MH844499). The ratio of the three opsins’ RPKM values (UV:B:LW) in the adult female head was 0.13:1:7.73 and in the adult male head was 0.13:1:7.85. The sum of the RPKM values of UV and B mRNA approximates to one seventh of the RPKM value of LW mRNA.

**Figure 3:**
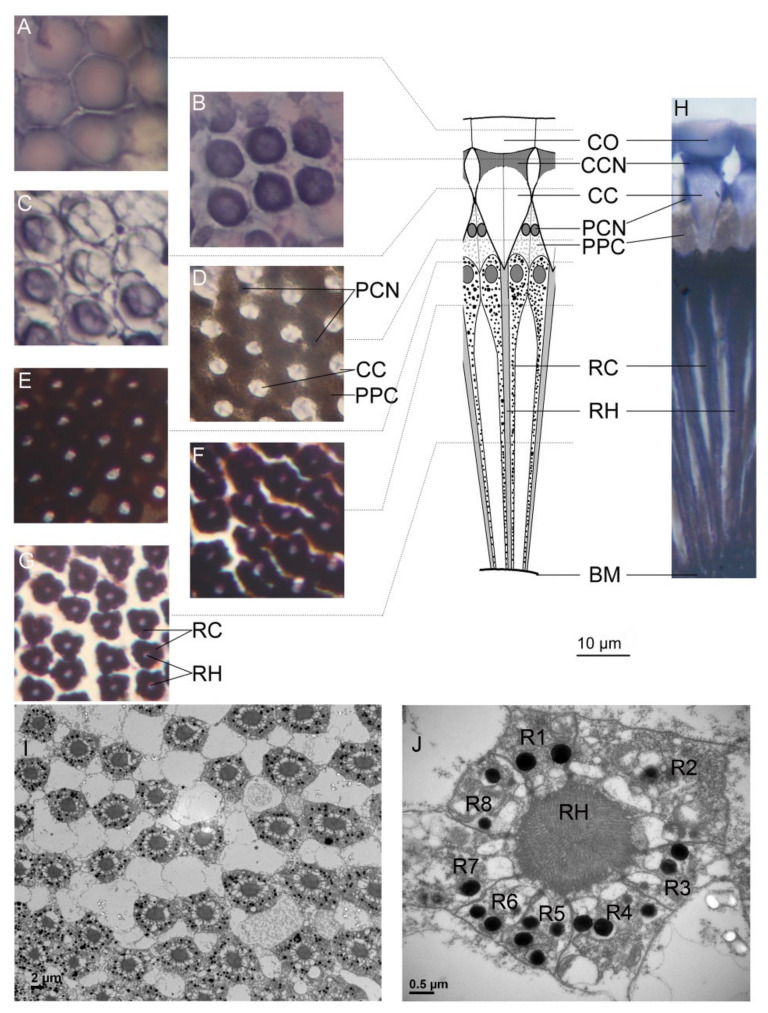
Fine structure of the ommatidium in *Empoasca onukii* adults: The transverse (**A**–**G**) and longitudinal (**H**) sections of ommatidia are shown with light micrographs, and the detailed transverse (**I**,**J**) sections of an ommatidium are shown with transmission electron micrographs. The dioptric apparatus of the ommatidium consists of the cornea (**A**) and the crystalline cone (**B**,**C**). The crystalline cones are surrounded by the primary pigment cells and are insulated from each other (**D**). In physical contact with the crystalline cone (**E**), the light-sensitive fused rhabdom is formed by eight retinular cells (**F**,**J**). The ommatidia are separated from each other in the middle section of the rhabdom (**G**,**I**). The fused rhabdom is formed by eight retinular cells, 1–8 (**J**). CO, cornea; CC crystalline cone; CCN, nucleus of cone cell; PPC, primary pigment cell; PCN, nucleus of primary pigment cell; RC, retinular cell; RH, rhabdom; R1–R8, retinular cells 1–8; BM, basement membrane. Scale bar = 10 μm in (**A**–**H**), 2 μm in (**I**), and 0.5 μm in (**J**).

Microstructural observation of compound *E. onukii* eyes showed that each ommatidium has two distinct structures: the dioptric apparatus, consisting of the corneal lens, crystalline cone cells, and primary pigment cells, and the photoreceptive layer, consisting of retinular cells and their rhabdomeres (Figure 3). Similar to *Callitettix versicolor* [31] and *E. vitis* [8], the adult *E. onukii* has an apposition type of compound eye because the crystalline cone directly connects to the rhabdom and the clear zone was not found. Each ommatidium contains eight retinular cells, which contribute parallel microvilli to form a centrally fused rhabdom (Figure 3I,J). The retinular cells also contain numerous screening pigment granules surrounding the rhabdom. In an ommatidium, adjacent retinular cells are connected by desmosomes and four tubular structures can be found near the four desmosomes in each rhabdom (Figure 3J). According to the organized positions of the four tubular structures, the retinular cell with two tubular structures on each side is numbered R1, the cell with no tubular structure on either side is numbered R3, and the remaining cells are numbered R2 and R4–R8 (Figure 3J) [12].

### 3.2. Spectral Sensitivity of the Compound Eyes

**Figure 4:**
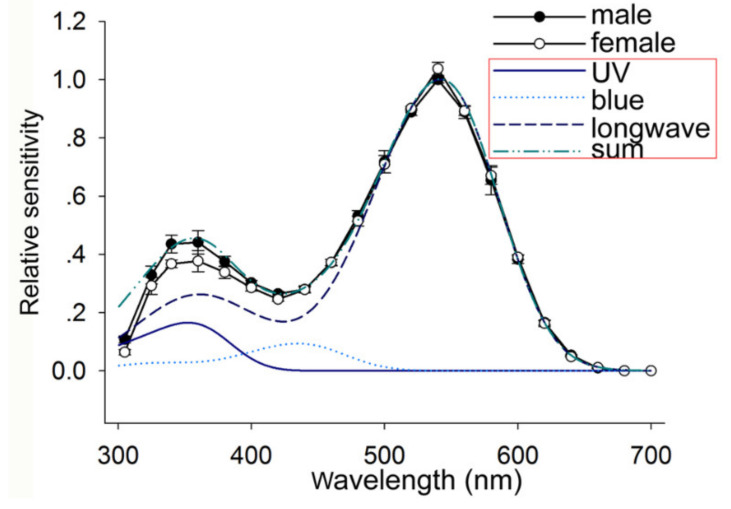
Spectral sensitivity curves of the compound eye of *E. onukii* measured with electroretinograms: The bidirectional recordings were typically repeated five times, yielding ten spectral scans. The open circles represent the spectral sensitivity of female adult leafhoppers, and the filled circles are that of males. The three blue fitting curves in the red frame represent the absorption spectra of putative visual pigments with peak wavelengths at 356 (UV), 435 (blue), and 542 (longwave) nm based on templates [32,33]. The dotted green color line in the red frame represents the absorption spectra of the summation of those three spectra at a ratio of 1:0.6:6.4 (UV/blue/green), normalized at 542 nm. UV, ultraviolet.

The spectral sensitivities of the compound eyes of *E. onukii* adults in both sexes were measured with ERGs. The spectral sensitivity curves of the compound eye have two major peaks, with the primary peak at 540 nm and a secondary peak at 355 nm (Figure 4). It was difficult to perform unicellular recording on each retinular cell because of the small body of *E. onukii*. Therefore, we used the visual pigment templates to predict the absorbance spectra of putative visual pigments. The formulae of Stavenga were applied when the peak wavelength was <400 nm [32], and the template from Govardovskii was applied when the peak wavelength was >400 nm [33]. Through the nonlinear least squares approach, the spectral sensitivity curves fitted well when the absorption spectra of three putative visual pigments had peak wavelengths at 356, 435, and 542 nm (UV, blue, and green receptors); the amplitudes of UV, blue, and green were in the ratio of 1:0.6:6.4, respectively (Figure 4). There was no significant sexual difference in the spectral sensitivity of the compound eyes of *E. onukii* adults (*p* > 0.05).

### 3.3. Reflectance Spectra of Host Foliage

Under illumination, the shapes of the reflectance spectra of tea leaves are remarkably consistent. Because of the photosynthetic pigments, the normalized reflectance spectra *R*(λ)*_i_* of tea leaves in the blue (475 ± 15 nm) and red (665 ± 15 nm) wavelength ranges are low, whereas that of the green (545 ± 15 nm) wavelength range is highest (Figure 1). All Pearson correlation coefficients among variables *R_i_*(*λ*)s were greater than 0.98, which indicates that there are significant linear relationships among these variables. When *R*_1_(*λ*) was used as the independent variable (*λ* ranged from 300 to 700 nm) and the other variables (*R_i_*(*λ*), *λ* = 2, 3…11) were regarded as dependent variables, the results from the linear regressions demonstrated that significant linear relationships existed between any two of these variables (*R* > 0.95, Table 1). However, the constant *b* did not equal 0; therefore, the actual ratios of the spectral wavelengths were different among these leaf samples and the hues of these leaves changed slightly and correlated with leaf age. The intensity of the whole reflectance spectra clearly decreased with increasing leaf age, and there was a significant linear relationship between *I_i_* and *i* (*R* = 0.82, Sig < 0.01).

### 3.4. Behavioral Experiments

**Figure 5:**
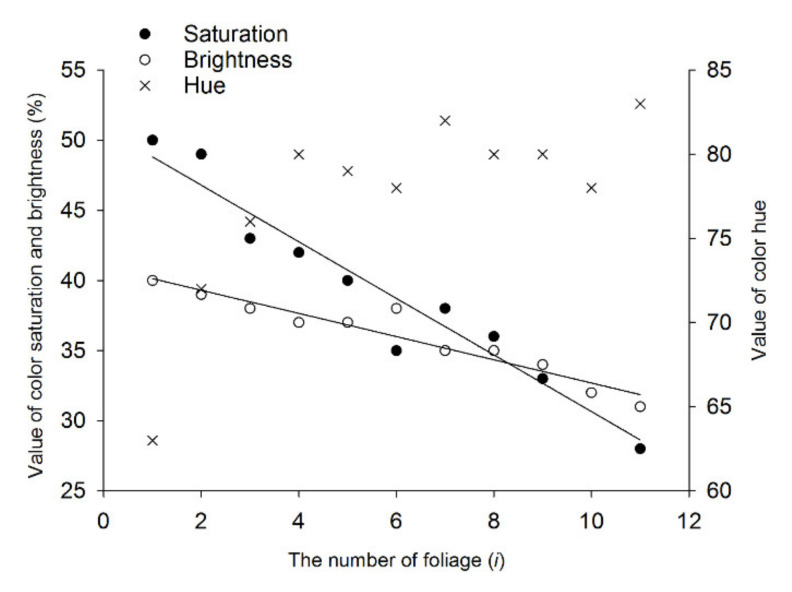
HSB parameters of tea leaves of different ages: On the obverse side of each leaf, on a total of six different branches, four sampling points were selected and measured. *H*, *S*, and *B* are foliage color hue (0–359), saturation (0–100), and brightness (0–100), respectively. The leaves of different ages are numbered with *i* (*i* = 1–11) from the top to bottom of a tea shoot (see Figure 1). Regression analysis of parameters *S* and *B* was conducted, with *i* regarded as an independent variable, to clarify the variation tendency of the two parameters with changing leaf age.

**Figure 6:**
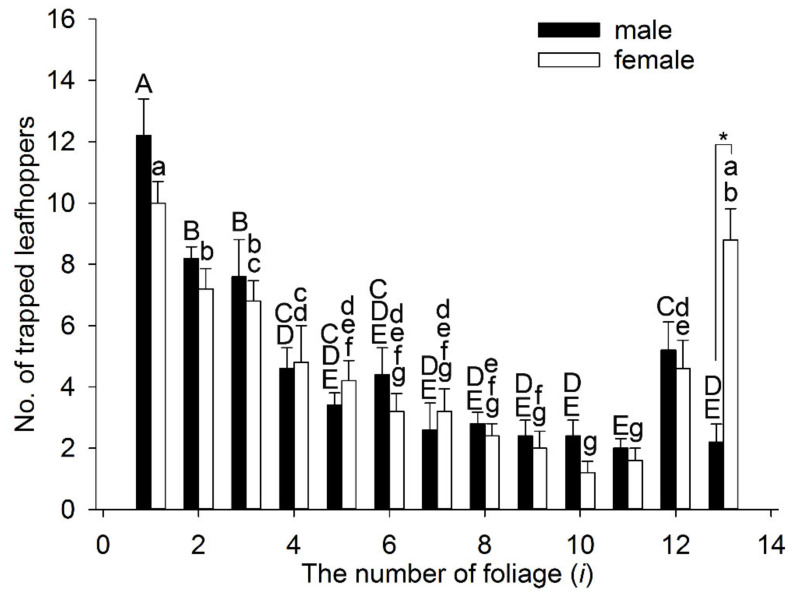
The first choice of different simulated colors by *Empoasca onukii* adults: Each experiment for males or females was repeated five times. Tea leaves of different leaf age are numbered with *i* (*i* = 1–11) from the top to bottom of a tea shoot (see detail in Figure 1). On the *x*-axis, the numbers (*i* = 1–11) are 11 simulated colors based on HSB parameters (see detail in Figure 5), the number 12 (*i* = 12) represents the control, and the blank space (*i* = 13) shows when leafhoppers had no choice of color. * A significant difference between the males and females (*p* < 0.05, independent-samples *t*-test). Mean values with the same letters are not significantly different (*p* > 0.05) within the male (uppercase) or female (lowercase) histograms, and those with different letters indicate a significant difference (*p* < 0.05, one-way analysis of variance).

Color parameters *H*, *S*, and *B* for all 11 test colors are shown in Figure 5. Color parameter *H* changes from 63 to 83 with increasing leaf age, which indicates that the hue of leaf changes from yellow to green. Color parameters *B* and *S* decrease as the leaf age increases, and the linear relations between *B* and *i* and between *S* and *i* are significant (*R*^2^ > 0.9), which indicates that the color of leaves gradually brightens with decreasing leaf age. As Figure 6 shows, the first choice of simulated colors of tea leaves by leafhopper adults also presents a regular pattern, i.e., with decreasing leaf age, the number of leafhoppers choosing the corresponding simulated color increases with significant differences (male, *F* = 16.889, *df* = 12, *p* < 0.05; female, *F* = 15.083, *df* = 12, *p* < 0.05). The number of leafhoppers of both sexes that chose the simulated colors was greatest for the first to third leaf below the bud (*i* from 1 to 3) compared with the simulated colors of the older leaves (*i* = 11). Compared with the control color (*i* = 12), there were more leafhoppers of both sexes that chose the simulated colors of the tender leaves (*i* from 1 to 3) but less of the older leaves (*i* from 9 to 11). Moreover, the number of females in the “no choice” bar (number 13) is significantly greater than the corresponding number of males (*t* = −5.618, *df* = 8, *p* < 0.05). The correlation analysis shows that the number of attracted leafhoppers has a significant positive correlation with *I_i_*, *S*, or *B* (*R* > 0.7, Table 2); that is, this tea leafhopper prefers brightly colored leaves with high saturation.

### 3.5. Discrimination of Host Foliage Color

The behavioral experiments indicated that *E. onukii* adults could discriminate the simulated foliage colors (obverse side) and chose the top leaves first without olfactory cues within a short distance (Figure 6). The chromatic contrast showed that, when the first leaf below the bud (*i* = 1) is regarded as the contrast object, Δ*S* of tea leaves *i* from 2 to 4 have a range from 1.118 to 1.458 and the Δ*S* values of leaves *i* from 5 to 11 have a range from 2.393 to 4.276. When the fourth leaf was used as contrast, only leaves *i* from 9 to 11 could theoretically be discriminated (Δ*S* > 2.3). The predictions of this model roughly match the result from behavioral experiments, i.e., foliage colors at the top (*i* = 1) could be distinguished from colors at the bottom by *E. onukii* adults, but foliage colors in the middle could not be distinguished from that at the top or bottom and may be intermediate colors for leafhoppers. The achromatic contrast showed that, when the top leaf (*i* = 1) was used as contrast, *C* values of tea leaves with *i* from 2 to 4 are all less than 0.08 (from 0.02 to 0.04), whereas the values for the other seven leaves range from 0.1 to 0.21 (i.e., *C* > 0.08). Moreover, the intensity of obverse sides of leaves (*i* from 5 to 11) could also theoretically be discriminated from the intensity of the fourth leaf (*C* > 0.08). We speculate that three different locations of the tea shoots, top (*i* = 1), middle (*i* from 2 to 5), and bottom (*i* > 5), can be discriminated based on color intensity by nearby *E. onukii* adults. Therefore, *E. onukii* adults mainly make their decision based on foliage intensity and not hues.

## 4. Discussion

Based on numerous references, some physiological characteristics of the compound eyes of *E. onukii* can be summarized. Similar to many hemipteran insects, such as *Callitettix versicolor* and *Nephotettix cincticeps* [12,31], the compound eye of the adult *E. onukii* is classified as the apposition type, with the ommatidia of *E. onukii* adults having eight photoreceptor cells and fused rhabdoms. Ommatidial heterogeneity is common in many insect species, such as butterflies [34,35], flies [36], bees [17], and some leafhoppers [12]. The transcriptome analyses showed that the sum of RPKM values of UV and B mRNA coincidentally approximates one seventh of the RPKM value of LW mRNA. Like *N. cincticeps*, which has two types of ommatidia composed of seven LW photoreceptor cells and one UV or B photoreceptor cell, it seems that ommatidial heterogeneity also exists in the compound eyes of *E. onukii*. For some insects with eight photoreceptor cells in each ommatidium, two photoreceptor cells have long axons that project directly to the medulla and may provide crucial input to the color vision system [12,18,37]. The two photoreceptor cells usually express UV and blue, UV and green, or blue and green sensitive opsins to form the trichromatic color vision system of insects [12]. For *E. onukii*, further determination of the spatial distribution of the three opsins in the ommatidia by double immunofluorescence labeling is necessary.

The expression of UV mRNA is significantly lower than that of B mRNA, but that has no effect on the sensitivity and tropism of *E. onukii* adults to UV light [38]. The opsin expression of some insects follows a circadian rhythm and can be impacted by physiological and environmental factors, such as starvation or photoperiod [39,40]. Spectral sensitivity can also be impacted by environmental factors (e.g., illumination), as was demonstrated for the Mantidae *Hierodula patellifera* (Serville) [23] and the Lepidoptera Sphingidae *Macroglossum stellatarum* (Linnaeus) [41].

The absorption spectra of LW pigments peaked at 542 nm, which is higher than that (~530 nm) of most reported insect species [13,34]. We previously found that *E. onukii* adults can be strongly attracted by yellow colors composed of the red and green spectra [9], which implied that *E. onukii* adults could recognize metameric colors and red light. Similar to *N. cincticeps*, the red-light discrimination of *E. onukii* adults may be attributed to the residual spectral sensitivity of green receptors in the long-wavelength region [12].

The number of females for “no choice” is significantly greater than the corresponding number of males, which indicates greater activity of males than females in the process of color choice. Actually, not only in the indoor experiments but also in the field experiments, the capture of male leafhoppers accounted for a dominant proportion on the color or light traps [42], which indicates that the activity of males may be greater than females in foraging or migration. In addition, male leafhoppers will actively search for the female in their mating process, such as *E. vitis*. Proper courtship begins only when the male locates the female [43], which will always keep still in most cases. Due to the limited time for indoor behavioral experiments, some females remained still and had not yet made a choice, which may be the reason for the significant difference.

Besides the *E. onukii* adults, a variety of herbivorous insects are known to be attracted to the color yellow [6,44]. However, “yellow” encompasses a range of different colors, not all of which can attract *E. onukii* leafhoppers; among the colors that do not attract leafhoppers are wheat (HSB: 69, 21, 87) and goldenrod (HSB: 43, 85, 85) [9]. The trends in variation of the HSB parameters show that younger tea leaves have higher saturation (*S*) and brightness (*B*). Developing leaves usually have a higher-than-average nitrogen content available in the plant sap and usually have weaker-than-average physical defenses on their surface [45]. *E. onukii* may prefer to inhabit the top or middle leaves because those leaves provide sufficient nutrients and are suitable for feeding or oviposition. It has been shown that the first landing choice was positively correlated with oviposition preference and larval survival, such as *Cicadulina bipunctata* [46] and *Amrasca biguttula biguttula* [47]. There are many special tea cultivars with diverse leaf colors, so these cultivars may have different susceptibility for *E. onukii*. The positive response of leafhoppers to the “super-normal foliage-type stimulus” [6] from the yellow color might be considered an active behavior associated with habitat location.

In response to feeding damage, some plants will release herbivore-induced plant volatiles (HIPV), which could directly deter herbivores or attract carnivorous arthropods as an indirect defense, such as lima bean (*Phaseolus lunatus*) [48]. The leaf volatiles released from tea plants can also enhance herbivore-associated defense responses and can influence the behavior of herbivores, such as (*Z*)-3-hexenol [49] or (*Z*)-(3)-hexenyl acetate [50]. Some secondary metabolites of tea plants can be induced by feeding damage of *E. onukii*, such as linalool, which is attractive to *E. onukii* adults [1,51]. In addition, some phytoplasmas may manipulate the behavior of its vector insect by changing the odor blend of its host plant [52]. For example, *Candidatus phytoplasma* can induce its host apple plants (*Malus domestica*) to release ss-caryophyllene, which could attract its vector insect *Cacopsylla picta* [53,54]. Therefore, the potential influence of phytoplasma or herbivore infection on the behavior of target insects should be considered in a further study on habitat location for *E. onukii*.

Vision of *E. onukii* leafhoppers plays a key role in their prealighting stage for host location. Beside the color of foliage, visual cues also include the leaf shape, relative size, motion, and background colors [5,6]. Pest trap-and-monitor methods based on color preference have been used for a long time [11,55], and further promotion of these methods requires studying the effect of the other abovementioned visual cues on habitat location of *E. onukii*.

## 5. Conclusions

Within the compound, apposition-type eyes of the adult *E. onukii*, each ommatidium has eight retinular cells, in which photoreceptors are distributed with peak spectral sensitivities at 356 nm (UV), 435 nm (blue), and 542 nm (green). Therefore, the adult *E. onukii* has the physiological ability to discriminate diverse colors.

For host foliage, changes in the spectral intensity and hue of reflectance light both correlate with leaf age, and a linear decrease of intensity with increasing leaf age was apparent. Without olfactory stimuli, adult *E. onukii* could discriminate colors and show preferences for the simulated colors of the obverse side of leaves at the top of tea shoots. Compared with foliage hues, the adult *E. onukii* could more easily discriminate host foliage of different leaf ages based on the foliage intensity.

## Figures and Tables

**Table 1:** The constants of the linear relationships between the normalized reflectance spectra *R*_1_(*λ*) and each *R_i_*(*λ*).

*i*	Equation Constants		
	*a*	*b*	*R*
2	1.125	−7.31	0.983
3	1.273	−18.315	0.978
4	1.279	−11.879	0.986
5	1.35	−19.891	0.991
6	1.232	−11.363	0.993
7	1.283	−17.695	0.99
8	1.307	−18.925	0.991
9	1.312	−19.929	0.987
10	1.357	−23.509	0.988
11	1.374	−26.361	0.99

*i* is the number of the leaves below the bud of different ages. In the linear equations, *R_i_*(*λ*) = *a R*_1_(*λ*) + *b*, the normalized reflectance spectra (*R_i_*(*λ*)) of the first leaf below the bud, was used as the dependent variable (*λ* ranged from 300 to 700 nm) and the other variables (*R_i_*(*λ*), *i* = 2, 3…11) were considered dependent variables. *R* is the Pearson correlation coefficient. All values were significant at *p* < 0.01.

**Table 2:** Constants of the linear relationships between *N_i_* and the normalized reflectance intensity (*I_i_*), color parameters *S* and *B* of the host foliage.

	Equation Constants			
**Male**	***a***	***b***	***R***	***Sig.***
*I_i_*	0.001	−12.975	0.701	0.016
*S*	0.411	−11.12	0.878	0
*B*	0.922	−28.408	0.817	0.002
**Female**	***a***	***b***	***R***	***Sig.***
*I_i_*	0.001	−11.306	0.722	0.012
*S*	0.375	−10.286	0.943	0
*B*	0.832	−25.705	0.866	0.001

*i* is the number of the leaves below the bud of different leaf age (*i* = 1–11). In the linear equations, *N_i_* = *a x* + *b*, the independent variable *x* was *I_i_*, *S_i_*, or *B_i_* and the dependent variable was the number of leafhoppers captured on each color (*N_i_*). *R* is the Pearson correlation coefficient.

## Supplementary Materials

The following are available online at https://www.mdpi.com/2075-4450/11/7/426/s1, Table S1: Primers used in opsin genes amplification.
